# Characteristics of brain activation in high-level football players at different stages of decision-making tasks off the ball: an fMRI study

**DOI:** 10.3389/fnhum.2023.1189841

**Published:** 2023-08-28

**Authors:** Ming-Hao Huang, Jian Lang, Ju Li, Zhe Qin, Ya-Ping Cao

**Affiliations:** ^1^School of Physical Education and Sports, Beijing Normal University, Beijing, China; ^2^Collage of Physical Education, Northwest Normal University, Lanzhou, China

**Keywords:** high-level football players, moving without the ball, decision-making, functional magnetic resonance, neural efficiency

## Abstract

**Objective:**

This study aimed to examine the neural mechanisms underlying the decision-making process of off-ball movements among high-level football players and ordinary college students, as well as the effect of long-term skill training on these neural mechanisms using functional magnetic resonance imaging (fMRI).

**Methods:**

The study recruited 20 professional college football players as the expert group (EG) and 20 novice football players with no background in sports-related disciplines as the novice group (NG). The participants performed the motor video observation and button-decision-making tasks, and fMRI data were acquired, pre-processed, and analyzed.

**Results:**

During the decision-making process regarding running without the ball, whole-brain fMRI scans were conducted on both the EG and NG. The analysis of these scans revealed noteworthy disparities in brain activity between the two groups. These disparities were observed during tasks involving motor video observation and button-based decision-making. According to the behavioral data, the EG made more correct decisions than the NG (*p* < 0.05); however, there was no significant difference in their reaction speed (*p* > 0.05). During video observation, both the EG and NG exhibited simultaneous activation in the frontoparietal cognitive area, primary somatosensory cortex, visual cortex, and insula. However, there were no significant differences between the two groups in terms of activated brain regions [false discovery rate (FDR) corrected to *p* < 0.05]. Regarding button-press decisions, the areas of the brain that were commonly activated in both the NG and EG were primarily located in the frontoparietal cognitive area, temporal cortex, and cuneus cortex. Notably, the left superior temporal gyrus, left inferior temporal gyrus, and left middle occipital gyrus exhibited greater activation in the NG compared to those in the EG (FDR corrected to *p* < 0.05).

**Conclusion:**

This study demonstrated that during motor video observation, the EG’s sports experience and professional knowledge can help them achieve better visual information processing strategies in specific areas of sports. During button decision-making, the EG was more economical, whereas the NG required more brain function activity to process visual information, confirming the “neural efficiency” hypothesis.

## Introduction

Football game scenes are constantly evolving, time and space are constantly changing, and physical confrontations are fierce. Athletes can influence their perception and understanding of a game scene through development of awareness of specific cognitions, effectively grasp key information, and make informed decisions while participating in sports (Murr et al., 2018). In football games, players are required to make numerous rapid decisions and reevaluate them based on the demands of the game (Vestberg et al., 2017). This process involves players’ creative decision-making, where their rich imagination and unexpected passing actions serve as effective behaviors for generating creative decisions, thereby significantly impacting the game (Fink et al., 2019). Furthermore, in football games, players spend a majority of their time off-ball, requiring them to rapidly accelerate, decelerate, and change direction during this process (Zouhal et al., 2019). For example, a pass-and-control team focusing on teamwork may find loopholes in the opponent’s defense through continuous running and observation, which inadvertently causes the opponent to suffer a “fatal blow.” Generally, high-level players must consider other players’ dominant areas, passing routes, and overall offensive or defensive development trends when making quick positioning decisions. In sports science, specialized perception is crucial to good motor decision-making and outstanding performance (Willams et al., 2006). Therefore, the decision-making level off the ball directly impacts team cooperation.

Research has established the relevance of “expert-novice” paradigms in motor cognition studies (Di Russo et al., 2005; Del Villar et al., 2007; Chang et al., 2011; Huang et al., 2018). A common application of this paradigm involves investigating the activation of cortical regions during executive decision-making. In this paradigm, exercise experts with extensive experience in exercise training are recruited as the experimental group, while a control group of novices is also included. The brain activity of both groups is analyzed during decision-making tasks. Several studies have demonstrated that experts exhibit reduced activation in specific brain regions during these tasks (Gobel et al., 2011; Guo et al., 2017; Blain et al., 2019). In a particular study, it was observed that expert archers exhibited activation of smaller and more focused neural networks compared to novice archers during an aiming decision-making task. However, novice archers demonstrated activation in a wider range of brain regions, including the primary motor cortex, primary somatosensory cortex, parietal cortex, prefrontal cortex, inferior frontal gyrus, and superior frontal gyrus (Kim et al., 2014). It has been identified that neural efficiency increases when cortical activation is reduced, and that reduced cortical activation can improve performance on tasks relating to expertise (Guo et al., 2017; Ludyga et al., 2020; Blazhenets et al., 2021). However, several studies have examined the effects of tasks on brain activation in athletes and found that decision-making tasks activate the cortex more than unfamiliar tasks (Sanchez-Lopez et al., 2014; Woods et al., 2014). The effect of differences in performance on a creative soccer task was found to be correlated with activity patterns in the left-lateral brain network, specifically involved in the processing of multimodal inputs from different sensory, motor, and perceptual sources; the middle temporal gyrus, central sulcus operculum, and cuneus are the main structures involved in this process (Fink et al., 2019). Based on these studies, it has been suggested that individuals with expertise in motor skills develop the capacity to generate preferential activation in brain regions associated with action planning and comprehension through prolonged training (Yang, 2015). Currently, there are two viewpoints regarding the study of the decision-making process. The first viewpoint refers specifically to the reasoning or judgment based on the task at hand (narrow definition). Researchers have found that the neural activity in the parietal cortex is related to the variables involved in the decision-making process (Platt and Glimcher, 1999). The activity of neurons in the parietal cortex is also correlated with the decision-making process in perceptual decision tasks performed by monkeys (Huk and Shadlen, 2005). These findings have revealed the neural mechanisms underlying decision-making in the narrow sense. The second viewpoint encompasses both perception (action observation) and judgment (broad definition). There is a close relationship between the observation stage and the final decision-making stage during the execution of decision-making tasks. Perception is defined as the process of recognizing, interpreting, and responding to information and stimuli (Correia et al., 2022). The observation stage typically involves perceiving and processing relevant information, including gathering and analyzing visual, auditory, or other sensory inputs. These inputs provide crucial information required for decision-making, such as available options, environmental conditions, and potential outcomes (Gold and Shadlen, 2007). Some scholars have investigated the neural mechanisms in the brain that are associated with environmental perception and understanding, revealing the importance of the brain’s integration of external environmental information and internal cognitive processes in the broad sense (Corbetta et al., 2008). Additionally, there have been studies focusing on the neural activity in the parietal cortex, revealing the temporal integration of visual motion signals in perceptual decision-making processes. Their findings have indicated that the neural activity in the parietal cortex is associated with the integration of visual information in perceptual decision-making (Huk and Shadlen, 2005). These findings support the broad viewpoint that the decision-making process involves not only perception but also the integration and judgment of information. Therefore, we divided the decision-making process into two stages, the video observation period and the button decision-making period, and assumed that the activation of brain regions of the two groups of individuals depends on the difference in their decision-making behavior.

Football players’ decision-making ability is based on their ability to read the game, which includes seeing the ball, teammates, opponents, and perception of what they are doing (Williams, 2000; Den Hartigh et al., 2018). During individual projects, neurones are equally active when an individual performs, imagines, or observes actions (Rizzolatti and Destro, 2007). Using “freeze motion” pictures, which capture motion snapshots in the middle of moving the objects, can provide implicit motion information, and functional magnetic resonance imaging (fMRI) studies (Kourtzi and Kanwisher, 2000; Senior et al., 2000) have demonstrated that implicit and actual movements share neural substrates. Furthermore, to gain experience and the ability to read the game effectively, football players require long-term professional training, making them an effective model for studying the effects of long-term training on specific tasks. Previously, the passing and receiving, shooting, and actions of the players were presented to the participants as video materials. This study focused on the movement of players without a ball, which is an important aspect of soccer tactics. Therefore, we used moving imagery as a surrogate for motor execution to examine functional differences in brain activity between different levels of expertise when watching a video and when faced with an off-the-ball decision-making task.

## Materials and methods

### Participants

A total of 40 participants took part in the study. Among these, 20 elite college soccer players from a top-tier university were included as the expert group (EG), consisting of 10 males and 10 females (age: 19.3 ± 1.1 years; all undergraduate students in their first to fourth year; average training experience: 8 years). Additionally, 20 novice soccer players with no background in sports-related disciplines were recruited as the control group (NG), including 10 males and 10 females (age: 19.9 ± 2.1 years; all undergraduate students in their first to fourth year; no professional training experience). At the time of the study, all specialist football players were active members of the varsity football team and were engaged in a regular training program. There was no historical specialty in any sport reported by the control group, except for weekly soccer activities during general soccer classes. The participants were all right-handed, and none reported a history of neurological or cardiovascular disease nor were they taking any medications known to affect their cognitive function. Prior to the study, all participants provided informed consent. The State Key Laboratory of Cognitive Neuroscience and Learning, Beijing Normal University Ethics Committee approved the study, and participants were compensated for their participation.

### Experiment task

Generally, using event-related design, different video stimuli are randomly presented during the experiment, and the timing of stimulus presentation is marked with time stamps. The time points of stimulus presentation can be used to differentiate brain activity during viewing periods from brain activity during decision-making periods. The purpose of this study was to compare activation responses to different decision-making tasks using fMRI experiments consisting of two different exercise conditions: movement observation and key-press decision-making. There were 10 trials in total, each consisting of two experimental conditions. Ten video games were presented as stimuli, programmed, and run using E-Prime Pro 2.0. First, a fixation point (“ + ”) preparation signal appeared on the screen for a duration of 8,000 ms, prompting the participants to focus their attention. Then, following the preparation signal, a game video was presented, with an average duration of 13,000 ms. After the video ended, participants were required to make key-press decisions based on the context and movement actions presented in the video. They were instructed to press the corresponding keys within a frozen frame lasting 2,500 ms to express their choices. Participants were tasked with making key-press selections within a limited time based on the prompts in the frame indicating the running direction of a highlighted team member [Direction 1 (Key 1), Direction 2 (Key 2), and Direction 3 (Key 3)]. Following the key-press decisions, a fixation point appeared for 2,500 ms to allow participants to return to a baseline state. This design facilitated a more stable magnetic field as well as avoiding interference between blood-oxygen-level-dependent signals generated by adjacent stimuli. The stimuli were presented in random order to avoid presenting identical stimuli.

**FIGURE 1:**
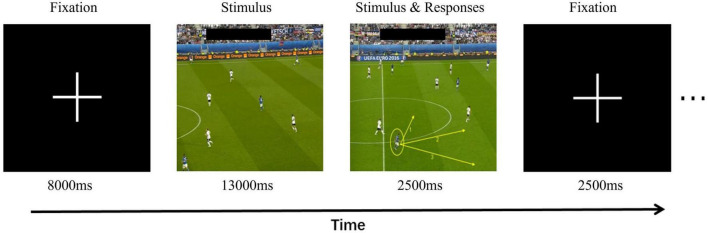
Experimental design for the decision-making task of running without a ball. This panel shows the time course of different stages of decision-making tasks off the ball.

The decision-making task design was adapted by referring to the standardized video task in soccer, whose objectivity, reliability, and validity was established in previous studies (Memmert et al., 2013). Our modification aimed to make a reasonable connection using the fMRI experiment, to enable participants receive specific instructions, stimulus presentation, duration, and feedback during the task. In this study video clips were presented from world-leading events such as the European Cup from a third-person perspective. The participants observed and analyzed the video from a third-person perspective. The information described from the third-person perspective was more abstract, whereas the purpose was clear (Libby et al., 2009). Three football experts with AFC A-level coaching certificates watched 10 video clips along with corresponding stimulus materials, and unanimously agreed that the material validity is reasonable. The stimulus materials included clips of a team in attack or defense and the subsequent movement options for the target player. The football experts were required to assess whether the correct movement direction of the target player, as provided in the stimulus materials, can facilitate or prevent favorable actions for our team in the upcoming offensive or defensive situations (Figure 1).

### fMRI data acquisition

Functional magnetic resonance imaging data were acquired using a Siemens Trio Tim 3T MR scanner equipped with a 12-channel phased-array receiver coil. It was designed to enable the participants to lie on their backs during the experiment. Sponge pads on the left and right sides of the head were fixed in the coil to reduce head movement. A projector connected to the computer projected the experimental content onto a screen in the imager. A single-shot gradient echo planar imaging sequence was used for functional imaging. The specific parameters were as follows: TR, 2,000 ms; TE, 30 ms; FOV, 200 × 200 mm; FA, 90°; and layer thickness = 4.32 mm. In parallel with the anterior commissure and posterior commissure, 33 slices of images were acquired at intervals covering the entire brain and the majority of the cerebellum. The scan matrix was 64 × 64 pixels, and the resolution was 3.4036 × 3.4036 × 4.3200 mm. This study also collected brain structure images of each participant to improve the image registration. A T1-weighted magnetization-prepared rapid gradient-echo sequence was employed, with the following scan parameters: TR = 1,900 ms, TE = 3.44 ms, and FA = 7°. This was a sagittal scan with an FOV of 256 × 256 mm and a pixel matrix of 192 × 256 pixels. There were 194 scanning layers, each layer was 1 mm thick, with no interval between layers. The resolution was 1 × 1 × 1 mm.

### fMRI data pre-processing and analysis

The experimental data were pre-processed using the SPM12 software. The pre-processing steps included the following: (1) data from the first 10 time-points were removed to stabilize the participants; (2) slice timing was carried out to reduce the error of inconsistent scanning time; (3) realignment occurred, excluding participants with head movement greater than 3 mm; (4) the T1 structural image was registered to the functional image, and the deformation field matrix was obtained by segmentation; (5) the deformation field matrix obtained in step 4 was used to standardize the functional image space in the Montreal Neurological Institute space; and (6) the image was smoothed using a smoothing kernel with a height and width of 6 mm to improve the signal-to-noise ratio.

### Statistical analysis

Using Microsoft Office Excel, the raw behavioral data was managed, and then SPSS 20.0 was used to analyze the valid data. Independent samples *t*-tests were conducted with physical activity level as the independent variable and key press accuracy and reaction time as the dependent variables.

The general linear model (GLM) implemented in SPM12 was used for first-level analysis, and t-statistic maps were generated as statistical parametric maps. The MRI model regressors for the observation phase and key press decision phase (with varying durations for each) were convolved with a typical hemodynamic response function. For second-level analysis, six motion parameters (6 × head motion) representing head movement in six directions were included as regressors in the design matrix to improve statistical sensitivity and obtain cleaner results. Contrast images representing the activation patterns in the brain during (i) the observation phase and (ii) the key press decision phase were used as inputs for multivariate pattern recognition analysis. In the result comparisons, independent samples *t*-tests were conducted to compare the NG and EG. A significance level of *p* < 0.05, corrected for false discovery rate (FDR), was used in the analysis.

### Correlation analysis

According to the peak coordinates of activated brain regions in two groups of participants at the whole-brain level during a key-press decision task, spherical regions of interest (ROIs) with a radius of 10 mm were created. Pearson correlation analysis was performed between the activation signals within each participant’s ROI and their behavioral performance data. A correlation coefficient close to 1 indicates a positive correlation, meaning an increase in brain region activity is associated with an increase in behavioral performance. A correlation coefficient close to −1 indicates a negative correlation, meaning an increase in brain region activity is associated with a decrease in behavioral performance. A correlation coefficient close to 0 indicates that there is little to no linear relationship between brain region activity and behavioral performance.

## Results

### Behavioral results

An independent sample *t*-test was performed on the rate of correct button decision-making and the response time of the EG and NG (Table 1). The results indicated that the EG had a significantly higher average accuracy than the NG [t (38) = −2.148, *p* < 0.05]; however, the average response times between the two groups did not significantly differ.

**TABLE 1:** Results of the button decision behavior test.

Group	*n*	Reaction time (s)	Accuracy (%)
NG	20	1.65 ± 0.28 s	42.5 ± 18.83
EG	20	1.63 ± 0.19 s	54.2 ± 14.81

The reaction time of the button decision in this table was measured during functional magnetic resonance imaging (fMRI) scanning. The values are expressed as mean ± standard error of mean. NG, novice group; EG, expert group.

### fMRI results

#### The activation of brain regions during video observation

**FIGURE 2:**
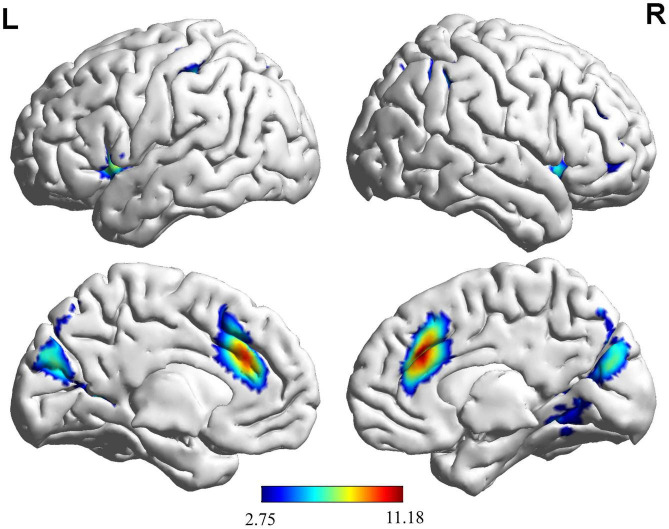
Brain activation maps for the EG and NG during motion video observation. This panel illustrates the voxel clusters where expert group (EG) and novice group (NG) interact during the task. The functional brain response is overlaid on a rendered standardized brain (MNI-space) with a threshold of 0.05 corrected only showing clusters with a minimum of 25 voxels, color scale represents the significant *t*-values. L, left; R, right; MNI, Montreal Neurological Institute.

Based on the results (Figure 2 and Table 2), we found that the brain activation areas for the NG during the motion video observation task were primarily located in the left insula, right medial and para cingulate gyrus, and right middle frontal gyrus. The brain activation areas for the EG during motion video observation included the left cuneus, left and right inferior temporal gyri, right orbital inferior frontal gyrus, left insula, left anterior cingulate and para cingulate gyri, and right triangular inferior frontal gyrus. The front parietal cognitive area, primary somatosensory cortex, visual cortex, and insula in the NG and EG were mainly activated during motion video observation. Regional centers were located in the cortex around the left distance fissure, left insula, right middle frontal gyrus, right medial and Para cingulate gyri, and left post-central gyrus. Furthermore, we conducted an independent sample *t*-test analysis of the brain imaging data of the two groups of participants; however, there was no significant difference between them (FDR corrected to *p* < 0.05).

**TABLE 2:** Activation of brain regions during motor video observation.

Group	Region	Cluster size	MNI coordinate			Peak T
			*x*	*y*	*z*	
NG	Insula L (AAL) (BA 13)	43	−42	9	−3	4.9774
	Cingulum mid R (AAL) (BA 9)	113	6	30	33	8.7376
	Frontal mid R (AAL) (BA 10)	29	39	36	21	5.0906
EG	Cuneus L (AAL)	112	−3	−81	24	8.0701
	Temporal inf L (AAL)	43	−48	−60	−9	5.1243
	Temporal inf R (AAL)	37	54	−54	12	4.5019
	Frontal inf orb R (AAL)	61	36	21	9	9.8385
	Insula L (AAL) (BA 47)	180	−36	18	−6	8.0985
	Cingulum ant L (AAL)	120	0	33	27	9.7568
	Frontal inf. Tri. R (AAL)	25	45	30	27	5.7513
	Parietal inf. R (AAL) (BA 40)	191	39	−48	45	6.5924
	Post-central L (AAL) (BA 40)	162	−48	−33	51	6.2817
EG and NG	Calcarine L (AAL)	122	−18	−54	6	7.33
	Insula L (AAL)	233	−33	15	−9	8.1406
	Frontal mid. R (AAL)	226	39	36	21	5.8986
	Cingulum mid. R (AAL) (BA 32)	103	3	30	30	11.1837
	Parietal inf. R (AAL) (BA 40)	222	45	−45	45	7.8783
	Post-central L (AAL)	154	−45	−30	48	6.5239

AAL, automated anatomical labeling; BA, Brodmann area; L, left hemisphere; R, right hemisphere; inf., inferior; mid., middle; orb., orbitofrontal; ant., anterior; tri., triangularis; MNI, Montreal Neurological Institute. False discovery rate (FDR) corrected to *p* < 0.05, with an extent threshold of 25 voxels.

#### Brain activation during button decision-making

**FIGURE 3:**
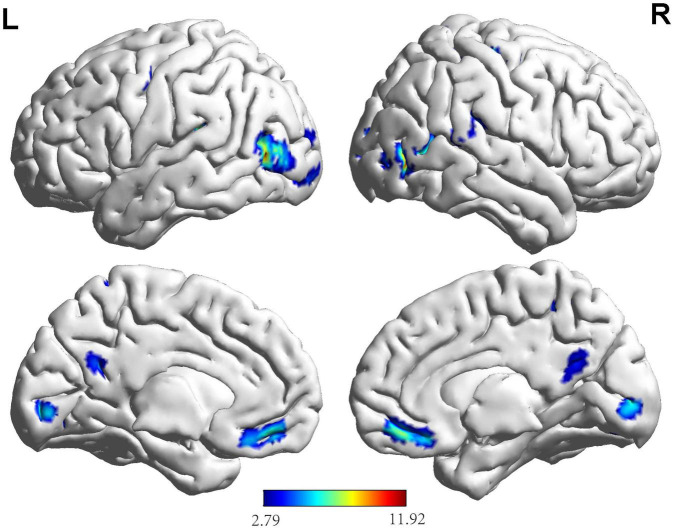
Brain activation maps for the EG and NG during key-press decision-making. This panel illustrates the voxel clusters where expert group (EG) and novice group (NG) interact during the task. The functional brain response is overlaid on a rendered standardized brain (MNI-space) with a threshold of 0.05 corrected only showing clusters with a minimum of 25 voxels, color scale represents the significant *t*-values. L, left; R, right; MNI, Montreal Neurological Institute.

Based on the results (Figure 3 and Table 3), the areas in the right temporal pole predominantly activated during the button decision-making task for the NG included the middle temporal gyrus, left rectus gyrus, left middle occipital gyrus, left middle temporal gyrus, right thalamus, left superior temporal gyrus, left precuneus, and left posterior central and superior parietal regions. The areas mainly activated in the EG included the left inferior occipital gyrus, right lingual gyrus, right middle temporal gyrus, and left middle temporal gyrus during the button-decision-making task. The areas of the brain that were jointly activated in the NG and EG during the button decision-making task were located primarily in the front parietal cognitive area, temporal cortex, and cuneus cortex. Most of the regional centers were located in the right rectus gyrus, right middle temporal gyrus, left superior temporal gyrus, left precuneus, left pre-central gyrus, right pre-central gyrus, right superior parietal gyrus, and the right dorsolateral superior frontal gyrus.

**TABLE 3:** Activation of brain regions during key-press decision-making.

Group	Region	Cluster size	MNI coordinate			Peak T
			*x*	*y*	*z*	
NG	Temporal pole mid R (AAL) (BA 21)	35	60	3	−18	6.575
	Rectus L (AAL)	61	0	42	−15	8.7819
	Occipital mid L (AAL)	302	−48	−75	3	13.728
	Temporal mid L (AAL)	72	−60	0	−18	4.773
	Thalamus R (AAL)	35	15	−27	0	9.2302
	Thalamus L (AAL)	31	−15	−30	0	7.5095
	Temporal sup. L (AAL)	93	−45	−36	−21	7.0448
	Precuneus L (AAL) (BA 31)	67	−3	−57	24	7.8132
	Pre-central L (AAL)	35	−48	−6	54	6.6802
	Pre-central R (AAL)	190	45	18	63	7.4431
	Parietal sup L (AAL)	146	−24	−51	57	7.3967
EG	Occipital inf L (AAL)	28	−24	−96	−3	4.9136
	Lingual R (AAL)	27	12	−81	−6	5.9148
	Temporal mid. R (AAL)	117	45	−66	6	9.7912
	Temporal mid. L (AAL) (BA 39)	52	−48	−72	9	7.1429
EG and NG	Rectus R (AAL) (BA 11)	43	3	45	−15	7.8059
	Temporal mid. R (AAL)	240	45	−63	6	11.9183
	Temporal sup. L (AAL)	56	−45	−36	21	8.3352
	Precuneus L (AAL) (BA 31)	33	−3	−57	24	5.7967
	Pre-central L (AAL) (BA 6)	36	−36	9	48	4.9758
	Pre-central R (AAL)	54	45	−21	63	6.6925
	Parietal sup. R (AAL)	53	21	−51	63	6.538
	Frontal sup. R (AAL)	37	18	−3	66	4.2839
NG > EG	Temporal sup. L (AAL)	226	−54	6	−6	6.4319
	Temporal inf. L (AAL)	32	−39	−21	−18	3.9251
	Occipital mid. L (AAL)	33	−24	−84	9	3.2539

AAL, automated anatomical labeling; BA, Brodmann area; L, left hemisphere; R, right hemisphere; inf., inferior; mid., middle; sup., superior; MNI, Montreal Neurological Institute; NG, novice group; EG, expert group; False discovery rate (FDR) corrected to *p* < 0.05, with an extent threshold of 25 voxels.

**FIGURE 4:**
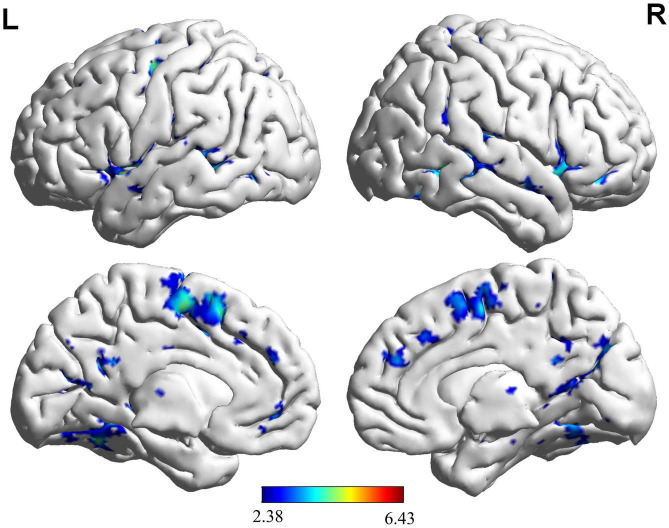
Brain regions activated more by the NG than the EG during key-press decision-making. This panel shows that novice group (NG) activates more clusters of voxels than expert group (EG). The functional brain response is overlaid on a rendered standardized brain (MNI-space) with a threshold of 0.05 corrected only showing clusters with a minimum of 25 voxels, color scale represents the significant *t*-values. L, left; R, right; MNI, Montreal Neurological Institute.

Based on the brain activation images of the two groups of participants, an independent sample *t*-test was performed, and age and sex were regressed as covariates. More brain areas were activated in the NG than in the EG during button decision-making tasks (Figure 4 and Table 3), mainly in the left superior temporal gyrus, left inferior temporal gyrus, and left middle occipital gyrus.

**FIGURE 5:**
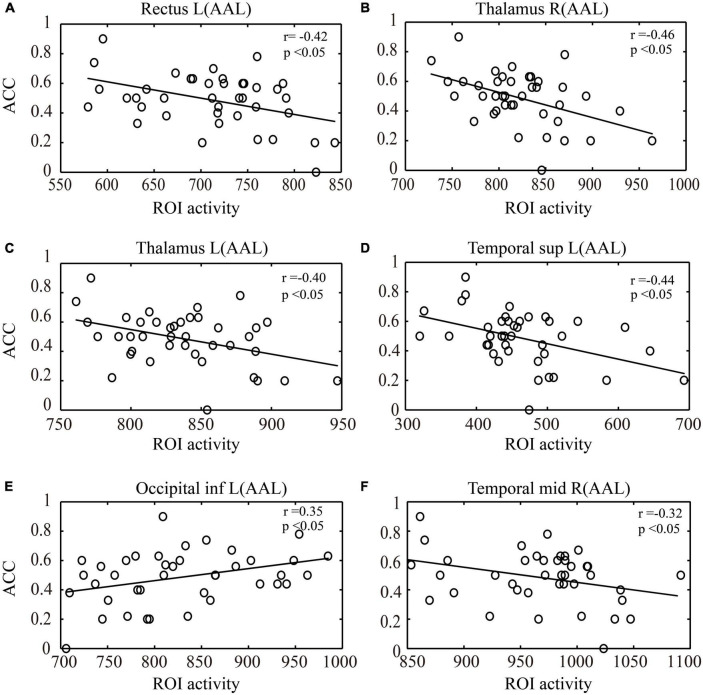
Correlation analysis between ROI activity during the key-press decision phase and key-press accuracy (ACC). **(A)** Correlation between activation in the left rectus gyrus and key-press accuracy. **(B)** Correlation between activation in the right thalamus and key-press accuracy. **(C)** Correlation between activation in the left thalamus and key-press accuracy. **(D)** Correlation between activation in the left middle temporal gyrus and key-press accuracy. **(E)** Correlation between activation in the left inferior occipital gyrus and key-press accuracy. **(F)** Correlation between activation in the right middle temporal gyrus and key-press accuracy. **(A–D)** represent the activated brain regions in the NG group, while **(E,F)** represent the activated brain regions in the EG group. ROI, region of interest; L, left; R, right; AAL, automated anatomical labeling; inf., inferior; sup., superior; mid., middle; NG, novice group; EG, expert group.

During the key-press decision phase, Pearson correlation analysis was conducted between the activated brain regions in the NG and EG groups and their key-press accuracy (Figure 5). The results showed that in the NG group, the activation of the left rectus gyrus (*r* = −0.42), right thalamus (*r* = −0.46), left thalamus (*r* = −0.40), and left middle temporal gyrus (*r* = −0.44) exhibited a significant negative correlation with accuracy (*p* < 0.05). In the EG group, the activation of the left precuneus (*r* = 0.35) showed a significant positive correlation with accuracy (*p* < 0.05), while the activation of the right middle temporal gyrus (*r* = −0.32) exhibited a significant negative correlation with accuracy (*p* < 0.05).

## Discussion

### Brain activation during motor videos

In both the EG and NG, the brain regions activated when viewing motion videos were similar, a finding that was consistent with many studies on moving images (Bakker et al., 2008; Oechslin et al., 2013; van der Meulen et al., 2014). In some studies which have explored the differences in brain activation between expert and novice groups during movement observation, the brain activation patterns of basketball, dance, and hand movement experts and novices during movement observation were investigated using techniques such as fMRI. These studies found that the neural activation patterns for both experts and novices during movement observation and execution were similar (Calvo-Merino et al., 2005; Aglioti et al., 2008; Cross et al., 2009). This suggests that even in experts, the brain mechanisms for observing and executing movements may be similar, possibly due to the automatization of movements and changes in body perception. In this study, the front parietal cognitive areas, primary somatosensory cortex, visual cortex, and insula showed high levels of activation. As a flexible cognitive control system, the front parietal cognitive area is activated during performing tasks that require cognitive control or executive functions (Vincent et al., 2008; Li et al., 2020). The post-central gyrus, located in the primary somatosensory cortex, plays an essential role in sensory processing (Menon et al., 2001). These regions were reported to be significantly activated in both the EG and NG, suggesting that football movement without a ball may require attention processing and sensorimotor integration (Corbetta and Shulman, 2002). It is estimated that players perform 150 sensorimotor responses per minute in the average adventure video game, which requires a coordinated effort between attention and sensory input (Gong et al., 2015). Considering that this study involved a visual information task, the activation of the visual cortex also indicated that it is a part of the cortex that processes visual information. In addition, the insula, particularly the left anterior portion, appears to play a key role in switching between brain networks (Yang et al., 2017). The results of this study demonstrated that significant activation is present in the left insula in both groups of participants, which may be related to the way participants process complex visual information.

It is noteworthy that the cuneus and inferior temporal gyrus were activated in the EG, but not in the NG. The occipital cortex, which includes the cuneiform lobe, plays a significant role in basic visual processes, attention, working memory, and response execution (Garavan et al., 2002; Booth et al., 2005; Matthews et al., 2005). In a study of table tennis players performing visuospatial tasks, the occipital cortex was also activated, suggesting that experts are required to process more visual information than novices (Guo et al., 2017). During complex sports, the brain’s perception system first receives stimulus information from the expert. Using the learned motor skills, the brain makes a final decision after identifying and matching tactical information with long-term memory. The inferior temporal gyrus lies outside the temporal cortex and is involved in cognitive functions, such as language, visual perception, and memory (Price, 2000; Noppeney and Price, 2002; Ojemann et al., 2002; Strakowski et al., 2005). Activation of the temporal cortex was previously shown to be modulated by the number of actions remembered (Cai et al., 2018). A study of half-pipe snowboarders found that the temporal cortex was significantly activated when they viewed their sport-specific images (Chen et al., 2020). Previous studies also demonstrated that experts developed specific perceptual-cognitive mechanisms to read higher-level physical cues more efficiently and effectively (Williams et al., 2003). Therefore, we speculated that experts’ sports experience and expertise may enable them to develop better visual information processing strategies in specific sports domains, enabling them to process visual information more effectively and eliminate irrelevant visual information. Considering that novices lack experience in related sports fields, they are unable to obtain effective visual information. Contrary with previous findings (Wang et al., 2008; van der Meulen et al., 2014; Zhang et al., 2018), neither EG nor NG showed any activity in the primary motor cortex. As the video was presented in the form of a general live television broadcast, the information provided to the participants consisted of the positions and movement trajectories of the players on the field and the movement trajectory of the football; therefore, no specific action was involved.

### Behavioral expression and brain activation during button decision-making

In respect of behavioral expression, the EG demonstrated higher accuracy than the NG during button decision tasks. In support of this finding, prior studies (Williams and Davids, 1998; Urgesi et al., 2012) demonstrated that athletes were more accurate in decision-making tasks and had better response stimulus discrimination and response selection abilities (Wylie et al., 2018). Considering the long-term training, elite athletes may be able to maintain stable, long-lasting, and accurate visual attention.

According to fMRI results, during the button decision-making process, experts and novices activated areas in the front parietal cognitive area, temporal lobe cortex, and cuneus lobe, in agreement with the results obtained during motion video observation. In addition, we observed bilateral activation of the pre-central gyri. It is evident that the pre-central gyrus serves many functions, the most important being motor function. The pre-central gyrus is responsible for movement execution by activating motor neurones (Piccinin et al., 2014). The EG and NG demonstrated hand button responses during the decision-making process. A previous study demonstrated that the pre-central gyrus is associated with movement planning (Indefrey, 2011). Movement planning was continued when a frozen picture was produced. An important aspect of motor planning is the interaction between different regions (Indefrey et al., 2000). These areas overlap with general cognitive areas, showing that decision-making is a collaborative process involving several domains.

In contrast to the NG, the EG showed reduced activation of relevant brain regions during the key-press decision-making stage. To explain this result, researchers used the “neural efficiency” hypothesis. With increasing skill levels, a decrease in neural activity in a particular brain region is referred to as neural efficiency (Neubauer and Fink, 2009; Karim et al., 2017). In other words, experts compared with non-experts, generally exhibit higher neural efficiency when performing tasks related to their area of expertise. In a study using fMRI technology to study central nervous system excitability by comparing the intensity of brain activity in pianists and control groups during finger motor tasks, pianists had lower activation in the primary motor cortex, supplementary motor area, and superior parietal cortex than participants in control groups (Krings et al., 2000). In another study, table tennis players showed less activity in task-related brain regions during on/off visuospatial tasks (Guo et al., 2017). In this study, it was observed that novices activated more areas, including the occipital and temporal lobe cortices. Sports cognitive psychologists believe that decision-making is the result of information, and that visual information is particularly important (Paul et al., 2016). An individual’s eyes are used to collect information and transmit details about their surroundings. The retina encodes the external visual world into various images, which are then transmitted by nerve impulses from the eyes to the visual center of the brain. In the occipital lobe of the cerebral cortex, this information is translated into “sights.” Similar to players in other ball sports, soccer players’ decision-making is influenced by their ability to identify shapes and colors in visual information, as well as the interaction between the musculoskeletal system and visual input. The ability to correctly interpret what is seen and done is an acquired skill, just as players move without the ball in a football game. As the structural basis for memory, the temporal lobe cortex is involved in higher cognitive behaviors and together with the occipital lobe participates in the processing of visual information. It can be concluded that compared to the efficient behavior of experts, novices require more time and energy to find available information and eliminate invalid data when performing the same task, resulting in more visual information systems being activated to process and analyze information, ultimately consuming more energy.

In the correlation analysis between the ROIs and behavioral performance (accuracy) in a key-press decision task, it was observed that in the NG group, activation in the left rectus gyrus, bilateral thalamus, and left temporal pole showed a significant negative correlation with accuracy during the key-press decision phase. The rectus gyrus is associated with episodic memory (Feng et al., 2020), the thalamus is involved in visual information processing (Shi et al., 2022), and the temporal pole plays an important role in perceptual processing (Bangert and Altenmuller, 2003). This finding suggests that the activation in these brain regions in the novice group may be associated with lower decision-making ability or uncertainty, leading to decreased accuracy in decision-making behavior. However, in the EG group, activation in the left cuneus showed a significant positive correlation with accuracy during the key-press decision phase, while activation in the right middle temporal gyrus showed a significant negative correlation. The cuneus is involved in visual information processing and visual spatial attention (Zhang et al., 2020), and increased activity in the right cuneus in the EG group may indicate better visual information processing ability compared to the NG group. The activation in the right middle temporal gyrus may be related to cognitive conflict in the decision task. The middle temporal gyrus is involved in visual processes, specifically in action observation (Cui et al., 2021). The presence of conflict or competition between different options during the decision process may lead to decision difficulty. Overall, these differences may reflect differences in cognitive strategies and neural resource allocation during the decision-making process between the experts and novices. Experts may have developed more optimized and efficient decision-making strategies through long-term training and experience, exhibiting more coordinated patterns of activation in specific brain regions. The lower accuracy observed in the NG during the decision task may be attributed to their limited understanding of task requirements and lack of experience in the decision-making process.

It is worth noting that the selective stimuli presented in video format in this study inevitably activates the visual cortex, which is one of the earliest regions in the brain to process visual information. When we perform motor tasks, such as playing basketball or soccer, the visual cortex receives visual information from the eyes, such as the position, speed, and direction of the ball. This information is then transmitted to other areas, triggering motor decisions. Decision-making is dominated by the areas of the brain responsible for developing and executing action plans. In motor cognition tasks, the decision-making stage formulates a motor plan based on information received from the visual cortex and transmits it to the motor execution area. In addition, the two stages of the decision-making task in this study are closely related. The motor video observation stage mainly involves sensory input and processing, while the key press decision-making stage involves decision-making and executing motor plans. In one study, it was found that the visual cortex is critical for the execution of motor cognition tasks during the observation stage. During visual perception tasks, the MT/V5 region (located in the occipital lobe of both hemispheres in the automated anatomical labeling template, including the inferior and superior temporal gyrus) receives visual input and transmits it to other areas, such as the frontal lobe, parietal lobe, and cerebellum, which are responsible for the decision-making stage to formulate motor plans (Tootell et al., 1995). Furthermore, some studies have also found a close relationship between the brain’s motor areas, such as the frontal lobe and motor cortex, and the visual cortex during the execution of motor plans. The activity in these regions plays a critical role in decision-making and executing motor plans (Cunnington et al., 2002; Passingham et al., 2010).

Based on cognitive neuroscience, this study revealed differences in the neural mechanisms of football experts and novices during sports video observation and decision-making. This study demonstrated that during the motion video observation process, the front parietal cognitive area, primary somatosensory cortex, visual cortex, and brain insula were activated in experts and novices; however, the cuneus lobe or inferior temporal gyrus was not activated in novices, indicating that experts’ experience and expertise can be used to improve their visual information processing strategies. In the button decision-making process, the left superior temporal gyrus, left inferior temporal gyrus, and left middle occipital gyrus were more activated in the NG than in the EG, which indicated that novices who performed the movement decision-making tasks required more brain function activities to process visual information than experts. These results can provide guidance for training and developing athletes, helping them better process and utilize visual information during competition, and improve their performance. In addition, these results can be applied to the fields of sports rehabilitation and sports medicine, providing new ideas and methods for rehabilitation training and treatment of sports injuries. Nevertheless, there are several limitations to this study. First, participants were not specifically divided and analyzed based on their positions on the field. Different positions and roles in a soccer game can have an impact on the decision-making process. For example, goalkeepers, defenders, midfielders, and forward play different roles and face different decision-making scenarios and pressures during a match. However, this study may have overlooked the influence of these unique characteristics of roles and positions on the decision-making process. Second, the use of video simulations alone to present game scenarios in the study may not fully replicate the dynamics and complexities of real matches. In actual games, athletes need to process real-time environmental information and observe and react to the behavior of other players. However, video simulations may not provide the same perception and response environment as in real games, thereby limiting the understanding of decision-making processes in real matches. Lastly, the fMRI technique used in the study has relatively low temporal resolution. In soccer games, decision-making processes are typically rapid and momentary, requiring athletes to make quick decisions and reactions. However, the temporal resolution of fMRI is generally within a few seconds, which may not capture the details and dynamics of such fast decision-making processes.

## Data availability statement

The original contributions presented in the study are included in the article/supplementary material, further inquiries can be directed to the corresponding author.

## Ethics statement

The studies involving human participants were reviewed and approved by the State Key Laboratory of Cognitive Neuroscience and Learning, Beijing Normal University. The patients/participants provided their written informed consent to participate in this study. Written informed consent was obtained from the individual(s) for the publication of any potentially identifiable images or data included in this article.

## Author contributions

M-HH, JiL, JuL, and ZQ conceptualized, designed, and conducted the experiments. M-HH, JiL, and JuL analyzed the data, interpreted the results, and wrote the manuscript. M-HH, JuL, ZQ, and Y-PC contributed to the conduct of the experiment and edited the manuscript. JiL and Y-PC revised the manuscript. All authors approved the submitted version.
